# Commentary: When the brain takes a break: a model-based analysis of mind wandering

**DOI:** 10.3389/fncom.2015.00083

**Published:** 2015-07-02

**Authors:** Vadim Axelrod, Andrei R. Teodorescu

**Affiliations:** ^1^The Gonda Multidisciplinary Brain Research Center, Bar Ilan UniversityRamat-Gan, Israel; ^2^UCL Institute of Cognitive Neuroscience, University College LondonLondon, UK; ^3^Department of Psychological and Brain Sciences, Indiana UniversityBloomington, IL, USA

**Keywords:** cognitive modeling, neuroimaging, pupillometry, mind-wandering, multivariate analysis

In recent years, the importance of integrating cognitive modeling with neuroimaging experiments has been repeatedly emphasized (e.g., Forstmann et al., 2011; Brown, 2014). The main challenge, however, remains realizing this approach in practice. Toward this aim, the recent work of Mittner et al. (2014), which explored the mechanisms of mind-wandering using a combination of brain imaging, pupillometry and cognitive modeling, serves as an important illustration of how this methodological amalgam can be effectively implemented.

In a functional MRI (fMRI) experiment with concurrent eye-tracking, the authors used a standard stop-signal paradigm (142 “stop-trials” and 284 “go-trials”). About 15% of the pseudorandom “go-trials” were followed by thought probes, requiring participants to rate their level of mind-wandering during the preceding trial. Subsequently, a Support Vector Machine (SVM) multivariate classifier was trained and tested on the thought-probe trials data using activity levels and functional connectivity within and between regions of interest (ROIs) of the default mode network (DMN) and its anti-correlated network, and measures of pupil size. Having successfully decoded the level of mind-wandering on thought-probe trials, the authors were able to use the classifier to predict the likelihood of mind-wandering on all remaining trials. Thus, the main benefit of using a combination of brain imaging, pupillometry, and behavioral measures over a typical behavioral experiment (e.g., Mcvay and Kane, 2012) was that it provided an estimation of mind-wandering levels for hundreds of consecutive trials, without the added distraction of incessant thought probes.

The authors' finding that pupils were informative for predicting the level of mind-wandering confirmed corresponding previous reports (Smallwood et al., 2012; Franklin et al., 2013), and anticipated an interesting recent study reporting a link between DMN activity and change of pupil size (Yellin et al., 2015). The results of Mittner et al. also accord well with brain stimulation (Axelrod et al., 2015) and fMRI imaging studies that implicated DMN in mind-wandering by using average activity level (for meta-analyses: Fox et al., 2015; Stawarczyk and D'Argembeau, 2015), multivariate (Tusche et al., 2014) and connectivity (Kucyi et al., 2013; Kucyi and Davis, 2014) patterns. Interestingly, while not discussed by the authors, the fact that the measures of functional connectivity, calculated using a sliding window of 40 s, contributed to successful prediction suggests that fluctuations in mind-wandering had a slow dynamics. To the best of our knowledge, slow fluctuations of mind-wandering have not been previously shown. Graphically, this phenomenon can be illustrated by plotting the predicted probability of mind-wandering across all trials as a function of time and by observing periodic oscillations.

In the second part of the study, the predicted likelihood of mind-wandering of all experimental trials was used in cognitive modeling analysis. Behavioral choice and Response Time (RT) data were analyzed using an independent race model where each available course of action (right, left, refrain from response) was represented by an independent stochastic accumulator. The first accumulator to reach a predetermined threshold determined the elicited response (or lack thereof) and its latency. The distributions of accumulator termination time were parameterized by seven parameters: three drift-rates with one for each response alternative; one common threshold; and three non-decision time parameters (one for each accumulator) representing the duration of all non-decision processes, such as perceptual encoding and response execution. Only three parameters were allowed to play a role in the mechanisms underlying the behavioral difference between trials with high- and low-level mind-wandering: correct drift-rate, refrain drift-rate and threshold (i.e., modulation of response caution). All three parameters were found to play a role and were lower for the high mind-wandering trials, reflecting poorer information processing efficiency and lower response caution.

Technical and theoretical assumptions are at the basis of every model and directly affect the interpretation of the fitting results (Teodorescu and Usher, 2013; Jones and Dzhafarov, 2014; Teodorescu et al., 2015). In this study a selective influence assumption (Ashby and Townsend, 1980) was made whereby mind-wandering influences only decision-related parameters (drift-rate and threshold). The grounds for this assumption, however, are not discussed. Importantly, mind-wandering might also affect non-decision processes. For example, by delaying initiation of perceptual encoding or habitual motor response (van Vugt et al., 2015). Thus, the selective influence assumption can hinder detection of any role that non-decision processes might play. To avoid potentially inaccurate estimates for the remaining (decision) parameters, the role of non-decision processes could be further tested by allowing this parameter to vary between conditions.

It is noteworthy that modeling interpretations are dependent not only upon selective influence assumptions, but also upon the choice of parameters that are omitted entirely from the model. For example, in order to provide adequate simultaneous accounts of both correct and error RT distributions, choice-RT models often include parameters that represent the magnitude of between-trial fluctuations in the state of the organism (Ratcliff and Mckoon, 2008). Although mind-wandering constitutes a potential source for such fluctuations, between-trial drift-variability parameters are absent in the current study. Furthermore, high drift-variability is associated with high behavioral response variability, which was indeed found by Mittner et al. Crucially, higher levels of between-trial drift-variability have been successfully used in the past to account for the higher response variability associated with mind-wandering (Mcvay and Kane, 2012). Thus, the inclusion of a drift-variability parameter could potentially provide an alternative interpretation for the modeling results of Mittner et al.

Finally, the stop signal paradigm used by the authors does not produce many error responses. The lack of error responses is a potential limitation since error-RT distributions are essential for constraining choice-RT models. Importantly, higher between-trial drift-rate variability, leads to slower errors compared to correct responses. Thus, the inclusion of error RT distributions could potentially disentangle the drift variability account of mind-wandering [higher between trial drift-rate variability] (Mcvay and Kane, 2012) from the [lower drift-rate + lower threshold] account adopted by Mittner and colleagues. The latter predicts increased error-rates, but a constant relation between correct and error RTs, whereas the former predicts increased error-rates and slower error RTs. In order to answer this question, future studies can consider using paradigms that generate more errors [e.g., Error Awareness Task (Allen et al., 2013)].

In sum, the study by Mittner and colleagues provides an inspiring example of combining cognitive modeling with neuroimaging—an approach that has great potential for advancing our understanding of cognitive mechanisms. It is noteworthy that the approach by Mittner et al. was to some extent indirect, since the brain imaging and pupillometry data were only used to generate per trial mind-wandering ratings, which were subsequently used in the modeling. Thus, a natural advancement would constitute a move toward an approach that directly models the neuroimaging data (e.g., Jahn et al., 2014).

## Funding

ART was funded by the Fulbright Scholar Program.

### Conflict of interest statement

The authors declare that the research was conducted in the absence of any commercial or financial relationships that could be construed as a potential conflict of interest.
